# Blind fish: An eye opener

**DOI:** 10.1093/emph/eoy020

**Published:** 2018-08-06

**Authors:** Akanksha Ojha, Milind Watve

**Affiliations:** Department of Biology, Indian Institute of Science Education and Research, Dr Homi Bhabha Road, Pune, India

**Keywords:** insulin resistance, Mexican cavefish, type 2 diabetes, comparative physiology

## Abstract

Lay Summary: Different species of vertebrates have conditions similar to human obesity, insulin resistance and type 2 diabetes. Increasing number of studies are now revealing that the causes and interrelationships between these states are substantially different in different species. Comparative physiology may turn out to be an eye opener for evolutionary theories of diabetes.

Obesity induced insulin resistance is believed to be central to type 2 diabetes. Recent work on Mexican cavefish, *Astyanax mexicanus*, has revealed a hyperglycemic phenotype similar to human type 2 diabetes but here insulin resistance is the cause of obesity rather than an effect. Instead of developing diabetic complications, the hyperglycemic fish lead a healthy and long life. In addition to fish, insulin resistance in hibernating bears, dolphins, horses, bonnet macaques and chimpanzees demonstrate that the relationship between diet, obesity, insulin sensitivity and diabetes is widely different in different species. Evolutionary hypotheses about type 2 diabetes should explain these differences.

Mexican cavefish, *Astyanax mexicanus*, a classic example of evolution in response to local conditions, has recently attracted attention because it exhibits a condition such as diabetes. The teleost fish has two distinct morphs: surface fish, which have fully developed eyes and dwell in open streams and cave dwelling fish, which arose from surface fish ancestors that got locked in a cave environment and in due course of time lost their vision as well as pigmentation [1, 2]. The blind fish are also larger compared with surface dwellers [1, 3]. Although these two morphs look so different, they are interfertile and can produce viable offspring [2]. The cavefish have multiple populations residing in different caves and have achieved the blind phenotype independent of each other. A loss of vision is accompanied by a series of behavioral changes related to feeding [4], mating [5], schooling [6] and loss of circadian rhythm [7]. 

The cavefish were found to be resistant to starvation and lost only half as much weight compared with surface fish after 2 months of starvation. This phenomenon was attributed to much higher body fat and low metabolic rate in cavefish [4, 7]. With normal feeding, some of the cavefish showed hyperphagia and accumulated more fat and had fatty liver compared with surface fish. Mutations in melanocortin 4 receptor (MC4R) were at least partly responsible for increased feeding, growth rate and starvation resistance in the cavefish [4]. Mutation in the same region of MC4R gene is associated with severe obesity in humans as well although the condition is uncommon [8]. Compatible with these observations is the finding that the cavefish are perpetually hyperglycemic, a condition that we generally recognize as diabetes and associate with obesity [3]. The story so far is compatible with the classical thrift-obesity-diabetes theory [9].

However, there are important twists and surprises in the story. We believe that in humans obesity causes insulin resistance, but in cavefishes, a P211L mutation in insulin receptor leads to insulin resistance and is at least partially responsible for the obese phenotype. When the mutant insulin receptor found in cavefish population was inserted in zebrafish, they also acquired the obese phenotype [3]. So here insulin resistance is primary, and obesity is the consequence. This observation is also similar to the result in mice that muscle-specific insulin receptor knockouts become obese [10]. This result is compatible with James Neel’s original idea that a gene responsible for diabetic tendency leads to obesity [9], but not compatible with the notion that obesity induces insulin resistance. However, insulin receptor mutations known in humans including the Donohue syndrome or the Rabson Mendenhall syndrome are non-obese and have post-prandial hyperglycemia but fasting hypoglycemia [11]. In the pathophysiology of type 2 diabetes, the cause-effect relationships are often unclear. Different schools of thought appear to propagate different and often contradictory causal relationships. The thought that obesity leads to insulin resistance dominated over a few decades and multiple mechanisms for obesity induced insulin resistance were proposed [8, 12–17]. Part of the evidence is based on correlations [18–23] and even the correlations are weak [24]. The old view that higher insulin response leads to obesity [9] is gaining grounds once again [25]. Furthermore, insulin resistance in the cavefish is not accompanied by increased levels of insulin meaning thereby that compensatory hyperinsulinemia does not inevitably follow insulin resistance [3]. Knocking out insulin receptors from muscle or fat tissue in mice also did not result in hyperinsulinemia [10]. In contrast, insulin receptor mutants in humans (Donohue syndrome or the Rabson Mendenhall syndrome) are hyperinsulinemic and have fasting hypoglycemia [11]. Thus there is no agreement on the causal relations between obesity, insulin resistance and hyperinsulinemia and causalities can be different in different species.

Chronic hyperglycemia in humans is believed to lead to a variety of complications which often take a lethal turn. However, the cavefish remain healthy and long-lived with their hyperglycemia. One of the main mechanisms of hyperglycemia induced complications is believed to be increased glycation of proteins [26, 27]. Among the independently evolved lines of fish, some appear to be relatively resistant to glycation, but others are not, and still, all of them are long-lived without any signs of diabetic complications [3]. It is likely therefore that either hyperglycemia is not the real cause of diabetic complications or it is possible to evolve multiple ways to avoid pathological consequences of hyperglycemia, if any. Compatible with this is the failure of clinical trials targeting strict glycemic control to arrest diabetic complications [28–31]. Therefore whether hyperglycemia is the predominant causal factor behind the pathological consequences of diabetes needs to be examined.

Cross species comparison is an important tool in evolutionary medicine, but so far data on insulin resistance in non-human species is uncommon. Although fragmentary, it is possible to put together data on insulin resistance in cave fish, black and grizzly bears (*Ursus thibetanus, Ursus americanus, Ursus arctos*) [32, 33], horses (*Equus ferus*) [34], chimpanzees (*Pan troglodytes schweinfurthii*) [35], bonnet macaques (*Macaca radiata*) [36] and dolphins *(Tursiops truncatus)* [37] to see the commonalities and differences. Hibernating animals exhibit a physiology that allows them to survive extreme temperatures by manipulating their metabolism. American and Japanese black bear revealed a seasonal variation in insulin sensitivity during summer/spring active period and fall/winter hibernating period [32, 33]. Grizzly bears undergo a similar annual metabolic cycle: in active period they gain weight up to 4 kg per day and lose as much as 50% of their body weight during for the 5–7 months of hibernation [38–41]. Females with higher fat content have higher fecundity and may produce more viable offspring [42, 43]. This seasonal fat deposition was seen to be a result of a changed insulin sensitivity in black bear as well as brown bear [32, 33, 41]. In summer, accompanying fat accumulation, the bears were more insulin sensitive. In contrast, insulin concentration was 2.5 times higher in hibernating grizzly bear, although they were euglycemic [41]. Interestingly when the adipocytes from hibernating grizzly bear were treated with serum from summer active grizzly bear, insulin sensitivity was partially gained by their adipocytes [41]. Contrary to humans, fat deposition appears to be associated with insulin sensitivity and fat loss with insulin resistance in bears.

Dolphins have also been shown to exhibit insulin resistance, but this is a response to high-protein diet [37]. Free ranging dolphins have lower plasma glucose and insulin levels compared with captive population, but both show increased insulin resistance with high-protein diet [37]. This observation contrasts the rodent models of high fat feeding to induce insulin resistance [44]. Insulin sensitivity in horses varies in different breeds [45] and decreases with age in mares, but there was no difference in mares fed with high sugar or high fiber diet [34]. In Chimpanzees and Bonnet macaques, insulin resistance is observed in a fraction of the population normally in the absence of any obesogenic intervention [35, 36]. Further in chimpanzees, the low social rank individuals were more insulin resistant than the high-ranking individuals [35] although the high-ranking individuals have greater access to food. The association of insulin resistance with food intake appears to be negative here.

Studies on different species of vertebrates seem to suggest that the relationships between food intake control, fat accumulation, insulin resistance, insulin production, hyperglycemia and its pathological effects can evolve differently in different species. It is not necessary that insulin resistance in all species follow obesity. Most evolutionary hypotheses for human type 2 diabetes stop at explaining obesity and assume that all other effects follow inevitably from obesity. This is not sufficient for an evolutionary hypothesis. If obesity induces insulin resistance in humans but not in all species, it is necessary to explain why this relationship evolved specifically in some species including humans. So far, the obesity centered evolutionary hypotheses about type 2 diabetes are far from satisfying this condition. The blind cavefish can prove to be eye-opener for evolutionary medicine.

**Conflict of interest**: None declared.
